# Gastrointestinal inflammation and cancer: viral and bacterial interplay

**DOI:** 10.1080/19490976.2025.2519703

**Published:** 2025-06-26

**Authors:** Valentina Arrè, Roberto De Luca, Stella Mrmić, Sofia Marotta, Stefano Nardone, Sandra Incerpi, Gianluigi Giannelli, Roberto Negro, Pankaj Trivedi, Eleni Anastasiadou

**Affiliations:** a Personalized Medicine Laboratory, National Institute of Gastroenterology “S. de Bellis”, IRCCS Research Hospital, Castellana Grotte, BA, Italy; b Department of Neurology, Beth Israel Deaconess Medical Center and Division of Sleep Medicine, Harvard Medical School, Boston, MA, USA; c Department of Clinical and Molecular Medicine, Sapienza Università di Roma, Rome, Italy; d Department of Medicine, Beth Israel Deaconess Medical Center and Division of Endocrinology, Harvard Medical School, Boston, MA, USA; e Department of Sciences, University “Roma Tre”, Rome, Italy; f Scientific Direction, National Institute of Gastroenterology, “S. de Bellis”, IRCCS Research Hospital, Bari, Italy; g Department of Experimental Medicine, Sapienza Università di Roma, Rome, Italy

**Keywords:** EBV, *H. Pylori*, SARS-CoV-2, microRNA, NLRP3, gastrointestinal disease, cancer, inflammation, immune escape

## Abstract

Gastrointestinal (GI) inflammation and malignancies arise from complex interactions between the host’s immune responses and microbial pathogens. Epstein–Barr virus (EBV), *Helicobacter pylori* (*H. pylori*), and Severe acute respiratory syndrome coronavirus 2 (SARS-CoV-2) contribute to chronic GI inflammation, immune evasion, and tumorigenesis through distinct but interconnected mechanisms. EBV, a widespread herpesvirus, establishes a latent infection in B cells and epithelial cells. It promotes gastric carcinogenesis through immune modulation, epigenetic changes, and viral microRNAs (miRNAs). *H. pylori*, a gastric carcinogen, induces chronic gastritis and gastric cancer (GC) through Cytotoxin-associated gene A (CagA) and Vacuolating cytotoxin gene A (VacA) virulence factors. These factors disrupt host immune responses and enhance oncogenic signaling pathways. Recent evidence also links SARS-CoV-2 to gut dysbiosis and inflammatory responses. It worsens immune dysfunction and hence potentially impacting GI pathology. EBV and *H. pylori* co-infections may synergistically amplify inflammatory signaling, creating a tumor-promoting microenvironment. This review emphasizes the molecular mechanisms by which these pathogens contribute to GI diseases, focusing on their immune evasion strategies and potential therapeutic targets. Understanding these interactions is essential for developing targeted interventions for infection-driven GI malignancies.

## Introduction

Viruses and bacteria play distinct roles in gastrointestinal (GI) pathology, influencing inflammation, immune modulation, and oncogenic transformation.^1^ Among these, EBV, *H. pylori* and SARS-CoV-2 are of clinical and biological interest due to their capacity to persist in host tissues, manipulate immune responses, and contribute to carcinogenesis. These pathogens affect different compartments of the GI tract and are associated with chronic inflammation, mucosal damage, and, in some cases, neoplastic transformation.^2^ EBV, frequently linked to hematolymphoid malignancies, also contributes to gastric lymphomas and epithelial tumors through its ability to evade immune surveillance and modulate host gene expression.^3–5^ *H. pylori* colonizes the gastric mucosa and is a well-established risk factor for gastritis, peptic ulcers, and gastric cancer.^6^ Although it primarily affects the respiratory system, mounting evidence shows how the intestinal epithelium sustains SARS-CoV-2 replication, ultimately affecting the GI mucosal immunity.^7^ This review discusses the roles of EBV, *H. pylori*, and SARS-CoV-2 in chronic gastrointestinal inflammation and immune evasion. We also explore the molecular mechanisms by which these infectious agents may contribute to developing lymphomas and epithelial gastric cancer. The significance of this review lies in its focus on the molecular interplay between these pathogens and the host, particularly in the context of chronic inflammation, immune evasion, and carcinogenesis. By dissecting the roles of EBV, *H. pylori*, and SARS-CoV-2, this review seeks to bridge existing gaps in knowledge, offering insights that could inform the development of targeted therapeutic strategies. Understanding these mechanisms is critical for identifying novel biomarkers and therapeutic targets, especially in an era where precision medicine and immunotherapy are at the forefront of cancer research.

## Mechanisms of EBV entry into GI epithelium and its role in inflammation, gastric carcinogenesis, and immune escape

### EBV infection of B-cells and epithelial cells

**Figure 1.**
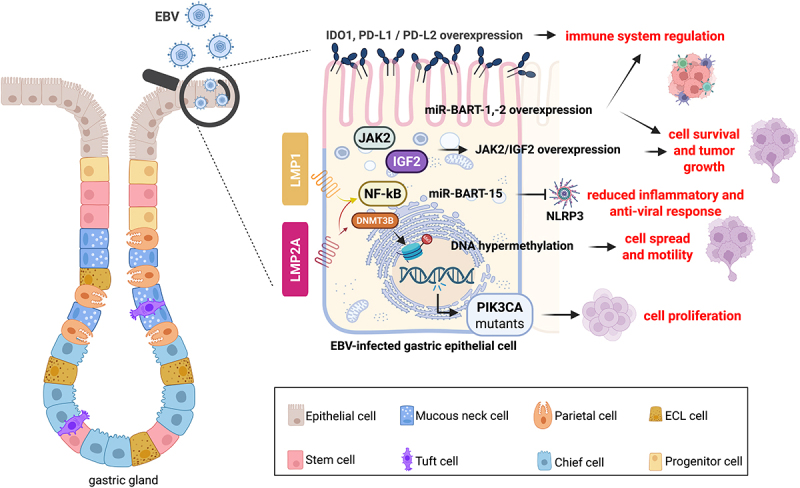
EBV infection of gastric epithelial cells impinges several molecular pathways leading to GC. EBV expresses latent membrane proteins-1 and − 2A (LMP1 and LMP2A) to maintain latency and cell proliferation through a positive feedback loop with NF-kB. LMP2A activates DNA methyltransferase 3 beta (DNMT3B) causing genome-wide methylation contributing to gastric cancer (GC). The virus may promote DNA hypermethylation in GC, with phosphatidylinositol-4,5-Bisphosphate 3-kinase Catalytic Subunit alpha (PIK3CA) mutations. These mutations may lead to cell proliferation. Other pathways leading to tumor growth, induced by EBV, are Janus kinase 2 (JAK2) and insulin-like growth factor 2 (IGF2) signaling. EBV-encoded microRNA (miRNA) clusters, including miR-BART-1 and − 2, are highly expressed and promote cell survival and immune evasion. In the tumor immune microenvironment (TIME), B cells infected by EBV were found to deliver exosomes carrying miR-BART-15 to epithelial non-EBV-infected cells. The virus might utilize the same mechanism to deliver miR-BART-15 into gastric epithelial cells to inhibit the activation of the inflammasome nucleotide-binding oligomerization domain, leucine-rich repeat-containing protein 3 (NLRP3), thus mitigating the inflammatory antiviral responses. EBV evades immune recognition by overexpressing the co-inhibitors indoleamine 2,3-dioxygenase 1 (IDO1), Programmed Death-Ligand 1 and 2 (PD-L1 and PD-L2).

EBV is a dsDNA herpesvirus that latently infects ~95% of people worldwide. Transmitted via saliva, it targets B lymphocytes by binding its glycoprotein gp350/220 to the Complement receptor type 2 (CD21) receptor, establishing latency that can disrupt B cell regulation and contribute to lymphomagenesis. EBV enters B cells *via* an endocytic pathway and fuses with the viral envelope and endosomal membrane through the interaction of glycoprotein 42 (gp42) with Human Leukocyte Antigens (HLA class II) and the core fusion complex glycoprotein H/glycoprotein L/gp42 (gH/gL/gp42).^8^ Besides B cells, the virus infects human epithelial cells of the oropharynx and gastric mucosa when swallowing saliva.^9,10^ Unlike B cells, epithelial cells generally lack CD21 or express only low levels, making them resistant to cell-free EBV infection.^10^ Several studies have proposed various models for the attachment of EBV to gastric epithelial cells.^11,12^ One of the most interesting mechanisms studied is cell-to-cell transmission. Co-culture experiments of EBV-positive B cells with epithelial cells indicated that EBV infection of epithelial cells is mediated by cell-to-cell contact. An increasing infection rate in epithelial cells suggests that EBV-infected B cells play a role in efficient viral transmission into the gastric epithelium. Cell-to-cell transmission may involve the formation of virological synapses (VS), which are tight adhesive junctions facilitating virus transfer between infected and non-infected cells without cell–cell fusion. This transfer infection is mediated mainly by CD11b^+^ memory B cells to epithelial cells at mucosal effector sites, such as the epithelium of the tonsillar crypts, most probably during the establishment of persistent infection of the host.^13^ Furthermore, these studies have suggested that the cell-to-cell transmission mechanism is intricately connected to the pathogenesis of gastric cancer (GC)^13^ (Figure 1).

### EBV latency in gastric epithelial cells and GI inflammation

EBV is known to establish latency within infected pre-malignant epithelial cells, leading to oncogenesis through its growth transformation-associated latent genes. Takada and colleagues successfully infected normal gastric mucosal epithelial cells with a recombinant EBV originally derived from the Burkitt lymphoma cell line Akata.^14–16^ The host epithelial cells exhibited a strong immune response to the viral infection through the secretion of Interleukin-1β (IL-1β) into the extracellular milieu. This pro-inflammatory cytokine is associated with *H. pylori* infection and considered an effector in the antiviral innate immune response. High levels of IL-1β were also observed in nasopharyngeal carcinoma (NPC) cases. All NPC cases are associated with EBV, and the viral latent gene expression is limited to the latency II pattern, characterized by Epstein–Barr virus nuclear antigen 1 (EBNA1) and latent membrane proteins (LMPs). Interestingly, NPC, EBV-infected gastric epithelium, and virus-positive gastric adenocarcinomas share the same pattern of latent viral gene expression. In NPC, EBV infection activates the NLRP3 inflammasome, which converts pro-IL-1β and pro-Interleukin-18 (IL-18) to mature IL-1β and IL-18 through Nuclear factor kappa-light-chain-enhancer of activated B cells (NF-κB) and a complex of several proteins that recognize the viral DNA in the cytoplasm.^17,18^ It is unknown whether a similar inflammasome activation mechanism may occur in the gastric epithelium after EBV infection. Other inflammatory factors include IL-6, IL-8, and tumor necrosis factor (TNF-α) in response to EBV infection.^19^ Additionally, the Heat shock protein 70 (HSP70) and NF-κB pathways were upregulated during the early stage of infection in a GC cell line, suggesting that the host response to viral invasion requires activation of these two pathways.^16^

Host cells react rapidly to EBV infection by activating immune and inflammatory pathways, forming the first line of defense. To establish latent infection in epithelial gastric cells, EBV must overcome robust host immune responses, which may explain the challenges in establishing EBV-infected cell lines using the “cell-in-cell” infection method. The above studies have raised questions about whether EBV is present in normal gastric mucosa. Ryan et al. estimated through quantitative polymerase chain reaction (qPCR) less than 1 EBV DNA copy per 100,000 cells in the normal gastric mucosa, comparable to the viral load observed in whole blood from healthy donors.^20^ Furthermore, EBV was present in a significant percentage of other gastrointestinal conditions, including 46% of gastritis lesions, 55% of Crohn’s disease samples, and 64% of ulcerative colitis samples.^20^ Immunohistochemistry (IHC) staining of EBV-positive biopsies from these lesions revealed the expression of EBV-encoded small RNAs (EBERs) in lymphoid cells. In contrast, the expression of LMP1 and LMP2A was undetectable by IHC. The lytic viral Recombinant EBV DNA polymerase processivity factor (BMRF1) and the EBV immediate-early protein BZLF1 (named *Bacillus amyloliquefaciens*, *B. amyloliquefaciens*, H one strain (BamHI) Z fragment leftward open reading frame 1) were present in the lymphoid cells of some ulcerative colitis tissues, which also had relatively high viral loads as measured by qPCR.^18^ The expression of lytic viral proteins suggests active viral replication in these lesions. These results indicate that EBV-infected lymphocytes are frequently found in the inflamed gastric and colonic mucosa. Other studies have demonstrated the accumulation of EBV-positive lymphocytes transmurally in ulcerative colitis.^21,22^ This type of lesion harbors T helper 2 (Th2) cells that produce Interleukin-5 (IL-5) and IL-6. Both cytokines are growth factors for EBV-infected lymphocytes, suggesting the possibility that the colon may be a potential site for EBV replication and transmission.^21,22^ The active viral replication and the strong host inflammatory responses observed in the proximal regions of the stomach, including cardia, fundus, and body, raise the possibility of a connection between EBV and the perpetuation of GI inflammation that may lead to GC^23^ (Figure 1).

Considering the prevalence of EBV in gastrointestinal lesions, a crucial question arises whether the virus actively contributes to gastric carcinogenesis or is it simply an incidental presence in inflamed tissue? While *H. pylori* is a well-known risk factor for gastric cancer, recent evidence indicates that EBV may promote gastric tumorigenesis on its own through mechanisms such as immune modulation, epigenetic reprogramming, and inflammatory signaling.^24^ Studies have demonstrated a lower prevalence of *H. pylori* infection in EBVaGC^+^ cases than in EBVaGC^−^ cases.^25^ For instance, Wu et al. reported *H. pylori* positivity in only 36.4% of EBVaGC^+^ cases as opposed to 68.3% in EBVaGC^−^ cases, while Luo et al. observed similar results (46.15% vs. 81.40%).^26,27^ These findings imply that EBV may function as an independent oncogenic driver in certain gastric cancers. Therefore, it is vital to distinguish between EBVaGC cases with and without *H. pylori* to enhance our understanding of disease heterogeneity and inform targeted therapeutic strategies.

### Pathways leading to EBV-associated GC

The gastritis-infection-cancer sequence of EBVaGC describes the molecular process of carcinogenesis starting with *H. pylori*-related gastritis and culminating in the development of EBVaGC.^28^ As mentioned earlier, almost 10% of GC cases are EBV-positive.^29^ This EBVaGC is most prevalent in Eastern Asia, Eastern Europe, and South America.^30,31^ Less than 10% of EBVaGCs are mucosa-associated lymphoid tissue (MALT) B-cell lymphomas.^5^ Prolonged immune activation and the production of cytokines may contribute to the development of gastric MALT. A study showed that EBV is involved in developing GC with a prominent lymphoid stroma, also known as lymphoepithelioma-like gastric carcinoma.^32^ EBV may impact the host genome in the epithelial cell via several mechanisms: a) Recurrent Phosphatidylinositol-4,5-Bisphosphate 3-Kinase Catalytic Subunit Alpha (PIK3CA) mutations involved in cell growth and division, b) DNA hypermethylation, which affects the regulation of genes involved in cell growth and division, c) Amplification of the Janus kinase 2 (JAK2) gene, involved in the regulation of cell growth and survival, potentially leading to abnormal cellular signaling, d) Overexpression of Programmed Death-Ligand 1 and 2 (PD-L1 and PD-L2, respectively) that play a role in immune system regulation.^33,34^

An integrated sequencing analysis of the genome, transcriptome, and epigenome of isogenic GC cell lines before and after EBV infection has been performed.^35^ The authors identified different signaling networks and EBV-encoding genes. LMP2A, expressed in approximately 40% of EBVaGC, was found to activate DNA methyltransferase 3 beta (DNMT3B), causing genome-wide methylation in EBVaGC.^35^ Using Chromatin Immunoprecipitation Sequencing (ChIP-seq), High-throughput Chromosome Conformation Capture chromosome conformation capture (Hi-C)-seq, and Chromosome Conformation Capture-on-Chip (4C)-seq datasets, a recent study has unraveled the repression of retrotransposon-competent human Long INterspersed Element 1 (LINE-1 or L1) by the viral DNA in EBVaGC, leading to an increased H3K9me3 methylation of L1.^36^ This mechanism might explain why EBVaGC displayed a lower frequency of copy number variation mutations than the EBV-non-associated GC (EBVnGC). Additionally, the study suggested that this difference in the mutation frequency may be related to a novel mechanism involving viral interference with the chromatin remodeling of the host genome. Another study on EBVaGC biopsies observed higher Insulin-like Growth Factor 2 (IGF2) expression in GC tissues compared to adjacent normal tissues. The results indicated that IGF2 gene promoter methylation and loss of imprinting (LOI) were significantly higher in EBVaGC tissues than in EBVnGC tissues. Thus, IGF2 may be involved in the pathogenesis of GC, and targeting IGF2 may be a potential therapeutic approach.^37^

A few studies have also investigated the role of individual latent viral proteins and viral miRNAs in GC. A positive feedback loop has been suggested between the oncogenic pathway NF-κB and LMP1 and LMP2A.^38,39^ This mechanism is essential for cell proliferation and maintenance of viral latency in EBVaGC. Furthermore, the EBV genome encodes for two clusters of miRNAs, miR-BART-1 and −2, derived from the BamHI A rightward transcripts. The miR-BARTs play a multifaceted role in manipulating host cell processes, promoting cell survival, immune evasion, and proliferation, and contributing to the altered metabolic characteristics of GC cells.^40^ Certain miR-BARTs, such as miR-BART7-3p and miR-BART9-3p, are the most highly expressed in EBVaGC clinical samples and may contribute to the oncogenic phenotype.^41^ A meta-analysis suggested that EBV-encoding miRNAs may serve as prognostic biomarkers in GC, with higher expression associated with poorer survival outcomes.^42,43^ As therapeutic interventions for EBVaGC patients, anti-viral agents such as nucleoside analogs like ganciclovir have been used to inhibit viral replication.^44^ Alternative anti-viral strategies include unmasking immunogenic viral peptides and infusing EBV-specific cytotoxic T cells to thwart the downstream effects of viral infection. Even though EBVaGCs have a cytosine phosphate diester guanine (CpG) island methylator phenotype (CIMP), there are few demethylating agents and ongoing clinical trials for this type of cancer. A Phase I clinical trial in 2017 using 5-azacytidine as a pretreatment before neoadjuvant chemotherapy yielded an overall responsive rate (ORR) of 67%, with 25% complete response in patients with resectable gastric and esophageal cancer (GC and EC, respectively).^45^ Recently, MC180295, an optimized cyclin-dependent kinase 9 (CDK9) inhibitor, has been found to inhibit the DNA repair and the cell cycle of EBVaGC cells.^33^ It is worth noting that PD-L1 and PD-L2 overexpression is relevant in immunotherapy, as targeting these proteins is a strategy to enhance the immune response against GC cells.^46^ For prevention purposes, developing vaccines against EBV might help mitigate the inflammatory responses associated with the virus in the context of ulcerative colitis and thus prevent cancer.^30,47^

### Immune escape strategies of EBV in GI inflammation and cancer

EBV is a highly successful virus which has managed to latently infect more than 7 billion people across the globe and since most EBV-infected individuals are immunocompetent, the virus has inevitably developed sophisticated immune evasion tactics. The infected host, in contrast, mounts an effective T and B cell response to viral antigens to keep it under check. Eventually, a host-viral equilibrium is reached, which ensures a lifelong latent infection. However, EBV is strongly associated with NPC and GCs, and it employs several immune evasion mechanisms, primarily to establish latent infection or transmission, but this most likely also facilitates tumor immune evasion. EBV expresses only a limited set of viral genes in cancers, minimizing the presentation of viral antigens and reducing the visibility of the infected cells to the immune system.^31^ Infected cells may downregulate Major histocompatibility complex (MHC) class I molecules, hindering the presentation of viral antigens to cytotoxic-T-lymphocytes (CTLs).^8^ EBV can exploit immune checkpoint (IC) pathways, such as the PD-1/PD-L1 axis. Moreover, interactions between PD-L1 on infected cells and PD-1 on T cells can lead to exhaustion and dampen the immune response. In the B cell lymphoma system, our group has shown that the virally encoded nuclear antigen, EBV virus nuclear antigen 2 (EBNA2), can induce the co-inhibitory IC PD-L1^47^ and downregulate the co-stimulatory IC, inducible T-cell co-stimulator ligand (ICOSL) expression^48^ by reducing miR-34a and increasing miR-24, respectively. However, EBNA2 is not expressed in EBV-positive epithelial cancers like NPC and EBVaGC. Given that functional redundancy is not uncommon in the viral world and that NPCs and EBVaGCs arise mostly in immunocompetent patients, it should come as no surprise if other viral proteins like EBNA1/LMPs and miRNAs are delegated with the task of interfering with ICs in these cancers.

It will be interesting to extend our findings in EBV-associated epithelial cancers to identify cellular miRNAs that regulate PD-L1 and ICOSL. Indeed, a recent article showed that T and Natural killer (NK) cell infiltration characterized the tumor immune landscape of EBVaGC and augmented IC expression, including PD-L1, Lymphocyte activation gene 3 (LAG3), Programmed Cell Death Protein 1 (PD-1), T cell immunoreceptor with Ig and the immunoreceptor tyrosine-based inhibitor motif (ITIM) domain (TIGIT), T-cell immunoglobulin and mucin domain 3 (TIM3), cytotoxic T-lymphocyte associated protein 4 (CTLA4), indoleamine 2,3-dioxygenase 1 (IDO1), and others.^49^ Furthermore, EBV-encoded miR-BARTs were negatively correlated with PD-1, CTLA4, TIGIT, Cluster of differentiation (CD) 69 (CD69), LAG3, B and T lymphocyte attenuator (BTLA), and TIM3, confirming their role as T cell exhaustion regulators in the Tumor immune microenvironment (TME). The miR-BARTs were also positively correlated with tumor cell proliferation.^50^ The increased expression of PD-L1 and IDO1 in the TME contributed to an inflamed T cell phenotype and the suppression of T cell proliferation. The landscape of a so-called “hot” immune TME makes EBVaGC patients good candidates for IC inhibitor therapies. Conversely, the same study correlated miR-BARTs’ high expression with reduced tumor immune infiltration, suggesting that miR-BARTs could mitigate the therapeutic potential of IC inhibitors. Another immune escape mechanism mediated through miR-BARTs has been described.^51^

In the context of B-cells, it was found that EBV miR-BART-15 targeted the Nucleotide-binding domain, leucine-rich – containing family, pyrin domain – containing-3 (NLRP3) inflammasome, enabling the virus to weaken inflammatory anti-viral responses through exosome delivery of the viral miRNA to non-infected cells. The underlying molecular mechanisms are not known. However, it will be interesting to study whether this viral miRNA has a similar role in GC (Figure 1). Deregulation of cellular miRNAs by EBV latent proteins in EBVaGC has been studied, albeit not in detail. Shinozaki et al. showed a synergistic effect of latency type I genes, such as B cell receptor-associated factor 0 (BARF0), EBNA1, and LMP2A in EBVaGC cell lines, leading to the downregulation of the miR-200 family and subsequent reduction of E-cadherin expression, which plays a crucial role in maintaining the integrity of epithelial tissues.^52^ One of the miR-200 family members, miR-200c-3p, targets PD-L1 in epithelial ovarian cancer (EOC). Reconstitution of miR-200c-3p in EOC cell lines reduced PD-L1.^53^ This implies that miR-200c-3p could be useful in restoring anti-tumor immunogenicity in EBVaGC as well. Another recently discovered and promising immunotherapy target is the integrin-associated protein (IAP) or CD47. Various tumor cells, including EBVaGC, expressed high levels of CD47. This interacts with the signal regulatory protein alpha (SIRPα) protein expressed on macrophages to avoid phagocytosis by sending a “do not eat me” signal.^54^ Blocking this interaction can increase the therapeutic efficacy of antibody-based anti-tumor therapies. Likewise, the combination of anti-CD47 and anti-PD-L1 antibodies enhanced the efficacy of immunotherapy against melanoma cells in a mouse model.^55^ A similar therapeutic approach for EBVaGC warrants consideration. Taken together, anti-IC approaches in combination with miRNA mimics and inhibitors hold promise for the development of RNA-aided immunotherapy.^56^

## *H. pylori* in GI mucosa inflammation and cancer and immune escape mechanisms

Infection by *H. pylori*, a spiral-shaped, Gram-negative bacterium, triggers chronic inflammation in the stomach, called non-atrophic gastritis, characterized by the aggregation of Cluster of differentiation 4^+^ (CD4^+^) lymphocytes and B cells in the gastric lamina propria.^57^ This inflammatory condition can lead to atrophic gastritis, GC, or MALT lymphoma if not cured. Over 50% of the human population is infected with *H. pylori*, which usually develops in pediatric children through saliva, stool, or vomit. The International Agency for Research on Cancer (IARC) has declared *H. pylori* as a type I carcinogen, and approximately 80% of GC cases are estimated to be associated with *H. pylori* infection. The driver genes involved in the pathogenesis of *H. pylori*-related GC are the virulence factors, namely, Cytotoxin-associated gene A (CagA) and Vacuolating cytotoxin gene A (VacA).^58^ Overexpression of CagA in transgenic mice induced gastrointestinal neoplasms, confirming its function as a bacterial oncoprotein.^59^ A comparative analysis in transgenic mice of CagA isoforms from Western and East Asian countries showed that western CagA-ABC is less oncogenic than E. Asian isoform ABCCC-type CagA. The differences were directly correlated with the number of EPIYA-C motifs in the CagA isoforms.^60^ In this context, it would be interesting to study whether these two different isoforms might be associated with a diverse microbiome between Western and Asian patients.

The pro-inflammatory cytokines related to *H. pylori* infection include Interleukin-1 α (IL-1α), which promotes angiogenesis, and IL-1β, which induces inflammation and is connected with NLRP3 inflammasome activation in *Hp*-associated gastritis and gastric carcinogenesis.^61,62^ Of note, single-nucleotide polymorphisms (SNPs) carried by IL-1β increase the risk of gastric cancer (GC) when *H. pylori* is present.^63^ Interestingly, the CagA-positive *H. pylori* strain activated the NLRP3 inflammasome and increased the invasion/migration of gastric cancer cells.^64^ High expression of the pro-inflammatory cytokine TNF-α was associated with peptic ulcer.^65–67^ In addition, in 2023 Dawson and colleagues reported a relationship between *H. pylori* and/or *Helicobacter felis* (*H. felis*) infection and Absent in melanoma 2 (AIM2) inflammasome-dependent IL-1β release, which contributes to the onset of gastric diseases.^68^

Detailed pathophysiological mechanisms for *H. pylori*-related carcinogenesis have been described.^69^ *H. pylori* impairs the host immune response and promotes its persistence within the host by adopting various strategies. One of these mechanisms is through the CagA, which plays a crucial role in regulating dendritic cells (DCs). Once inside the antigen-presenting cell (APC), CagA is phosphorylated, leading to the activation of Sarcoma virus (Src) homology two domain-containing protein tyrosine phosphatase-2 (SHP-2), which suppresses the enzymatic activation of TANK-binding kinase 1 (TBK1). It inhibits interferon regulatory factor-3 (IRF-3) phosphorylation through TGF-beta-activated kinase 1 (TAK1) binding protein 1 (TAB1) and its nuclear translocation, reducing interferon production by DCs. The bacterium proliferates within DCs, impairing their function by inhibiting the production of the pro-inflammatory cytokine Interleukin-12 (IL-12). The inhibition of DC maturation and IL-12 secretion, along with increased anti-inflammatory Interleukin-10 (IL-10) production, leads to suboptimal T helper 1 (Th1) development and activation.^69^

**Figure 2.**
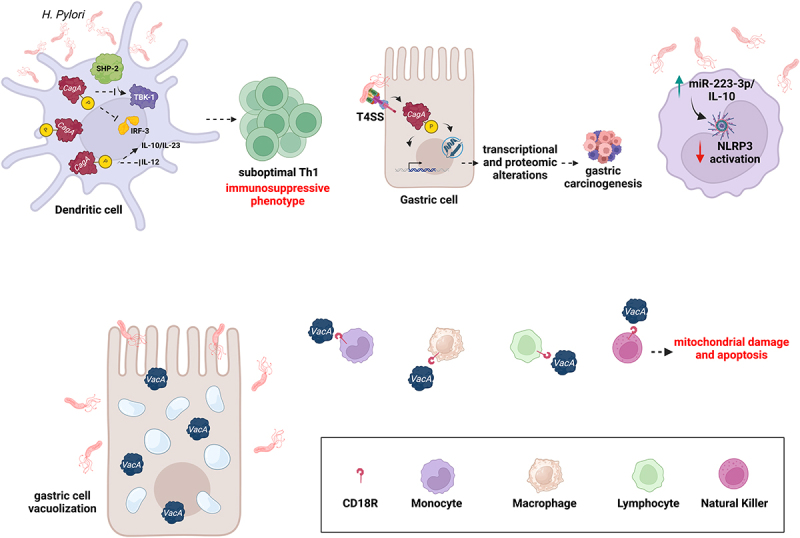
*H. pylori* strategies against host immune response. *H. pylori* infects the dendritic cells (DCs) and expresses cytotoxin-associated gene a (CagA) oncoproteins which undergo phosphorylation and activate src homology two domain-containing protein tyrosine phosphatase-2 (SHP-2). This activation suppresses TGF-beta-activated kinase 1 (TAK1) binding protein 1 (TBK-1) activation, inhibiting interferon regulatory factor-3 (IRF-3) phosphorylation and nuclear translocation. Consequently, interferon production by DCs is reduced. The bacterium proliferates within DCs, impairing their function by inhibiting the production of the pro-inflammatory cytokine IL-12. On the other hand, CagA can increase the anti-inflammatory IL-10 production, resulting in suboptimal T helper 1 (Th1) development and activation. The CagA protein is delivered into gastric epithelial cells through the bacterium’s type IV secretion system (T4SS). Once inside the host cells, CagA becomes tyrosine-phosphorylated by host kinases and interacts with various host signaling pathways, leading to altered genomic instability, inflammation, and gastric cancer. *H. pylori* virulence factor Vacuolating cytotoxin gene a (VacA) causes vacuolization in epithelial cells of the gastric mucosa, binds to the Cluster of differentiation 18 CD18 receptors of immune cells like monocytes, macrophages, polymorphonuclear lymphocytes (PMNs), and natural killer (NK) cells, inducing mitochondrial damage and apoptosis. *H. pylori* infection of a monocyte/macrophage human cell line, named THP-1, induced miR-223-3p and IL-10 expression to regulate the activation of nucleotide-binding oligomerization domain, leucine rich repeat and pyrin domain containing 3 (NLRP3) inflammasome. This mechanism fine-tunes the host inflammatory response, preventing excessive inflammation and offers novel insights into how *H. pylori* establishes and maintains chronic infections.

Long-term infection with *H. pylori*, especially strains carrying the CagA gene, results in an increased expression of PD-L1 and increased production of immunosuppressive cytokines such as IL-10 and IL-23 by DCs (Figure 2). CagA protein is translocated into gastric epithelial cells via the bacterium’s type IV secretion system (T4SS). This process involves the direct injection of CagA into host cells, where it undergoes tyrosine phosphorylation and interacts with various signaling pathways, contributing to cellular alterations associated with pathogenesis.^70^ In addition, VacA, which causes vacuolization in epithelial cells of the gastric mucosa, binds to CD18 receptors of immune cells like monocytes, macrophages, polymorphonuclear lymphocytes (PMNs), and NK cells, inducing mitochondrial damage and apoptosis.^71^ Pachathundikandi et al. showed that infection of the THP-1 monocyte/macrophage cell line and peripheral blood mononuclear cells (PBMCs) from healthy donors increased the levels of miR-223-3p and IL-10 to regulate NLRP3 expression and activation.^72^ This cooperative mechanism likely represents a fine-tuned control mechanism to balance the inflammatory response, preventing excessive inflammation and allowing the immune system to respond to the infection.

Recently, Koch et al. demonstrated that CD8^+^ tissue-resident memory T cells (TRM cells) play a crucial role in early immune responses to *H. pylori* by targeting the CagA oncoprotein. These cells infiltrate the gastric mucosa and perform antigen-specific effector functions but are gradually supplanted by CD4^+^ T cells during chronic infection. This shift promotes pro-inflammatory cytokine production, leading to mucosal injury, epithelial disruption, and neoplastic transformation.^73^ Together, these findings underscore how *H. pylori* subverts host immunity to ensure its persistence and reshapes the gastric immune landscape in ways that fuel chronic inflammation and drive gastric carcinogenesis (Figure 2).

## Mechanisms underlying *H. pylori*-related gastritis and EBV infection in GI inflammation and cancer

There are conflicting findings regarding the relationship between EBV and *H. pylori* co-infection in gastric precancerous diseases, such as atrophic gastritis and GC. On the one hand, a negative correlation has been proposed between EBV and *H. pylori* infection, suggesting that in GC, the presence of one pathogen might inhibit the colonization or the functioning of the other, potentially leading to antagonistic effects.^74,75^ In contrast, another study suggested a positive correlation between EBV and *H. pylori* co-infection.^76^ This *scenario* is associated with severe inflammation and an elevated risk of progression to intestinal-type GC and gastric precancerous diseases. The order in which *H. pylori* and EBV infections occur in cases of co-infection can vary, and the precise timing may not always be clear. Three cohort studies report that roughly 10–46% of EBVaGC patients showed evidence of current or prior *H. pylori* infection, suggesting that while co-infection occurs, EBVaGC often arises independently of *H. pylori*.^25–27^ The order of infection may depend on various factors, including the individual’s exposure to these pathogens, their immune status, or environmental factors. Both infections can persist for a long time (EBV for a lifetime) and the interactions between the two pathogens in the gastric mucosa are complex and not yet fully understood.

A study of over 300 pediatric patients found that EBV and *H. pylori* co-infection (HP+/EBV+) was linked to severe gastric lesions, with over 80% of the cases with severe mononuclear (MN) and moderate-severe PMN lymphocyte infiltration occurring in co-infected patients.^77^ This shows that EBV seropositive individuals outnumber those encounter*ing H. pylori* infection during childhood. The study showed that single infections with *H. pylori* CagA+ strains are associated with mild-to-moderate infiltration. However, co-infection with EBV leads to more severe damage to the gastric mucosa. A hypothetical *scenario* could be the interaction between the transcription factor Zebra trans-activator (Zta), a master regulator of the EBV lytic cycle, and the bacterial oncoprotein CagA. The latter can induce protein kinase C (PKC), which then induces Zta, and thus the EBV lytic cycle. CagA triggers a loss of polarity in epithelial cells that might favor EBV tropism for this cell type. So, in a two-pronged approach, while *H. pylori may* favor severe inflammation, EBV, through its latent transforming genes and their oncogenic potential, may add “insult to injury” in gastric lymphoepithelial mucosa.

The virally encoded latent proteins LMP1, LMP2A, and the *H. pylori* protein CagA induce common oncogenic pathways, such as NF-kB and mitogen-activated protein kinases (MAPKs), which may lead to gastric epithelium transformation. In co-infected patients, pro-inflammatory IL-8 and IL-1β expression levels were increased^77^ (Figure 2). Kashyap et al. showed that co-infection of GC epithelial cells may lead to a more favorable environment for both pathogens and a higher pathogen-associated gene expression.^78^ In particular, coinfection of GC cells increased expression of EBV genes, EBNA1 and EBV nuclear antigen 3C (EBNA3C), and *H. pylori* genes, such as CagA, the blood group antigen binding adhesin (babA), and 16S rRNA. Furthermore, the interplay between EBV and *H. pylori* led to the dysregulation of proteins involved in: 1. cell cycle progression, like cyclin D1, death-associated protein kinase 3 (DAPK3), proliferating cell nuclear antigen (PCNA), and Protein kinase B (AKT); 2. cell migration. Moreover, EBV and *H. pylori* co-evolution altered the expression of the tumor suppressor retinoblastoma protein (pRB), the phosphatase and tensin homolog (PTEN) protein, the oncoprotein gankyrin, the antiapoptotic B-cell leukemia/lymphoma 2 protein (Bcl2) and the tumor protein Tumor protein P53 (p53), critical for enhanced carcinogenic activity.^78^ This information may support the development of targeted therapeutic strategies for preventing or treating gastric cancer associated with these infections.

It is also essential to examine useful biomarkers, especially miRNA dysregulation, to identify malignant developments. Evidence suggests that both pathogens downregulate the let-7 and miR-200 families, leading to increased inflammation and the induction of the Epithelial–Mesenchymal Transition (EMT).^79^ Furthermore, both pathogens increased miR-155 expression, suppressing the release of pro-inflammatory cytokines and contributing to persistent infection and oncogenesis. The crosstalk between pro- and anti-inflammatory miRNAs induced or suppressed by these pathogens results in the maintenance of persistent infection creating a microenvironment conducive to the oncogenic transformation of epithelial cells. The overall effect of this miRNA regulation might generate a microenvironment that drives the oncogenic transformation of epithelial cells.^80^ The regulation of miRNAs by pathogens is a complex and evolving field of study, and a better understanding of these interactions may provide insights into the molecular mechanisms underlying infectious diseases and their association with cancer development.

## SARS-CoV-2 fosters a gut dysbiosis phenotype and alters the metabolome profile in infected subjects

Although Coronavirus disease 2019 (COVID-19) disease, caused by SARS-CoV-2 infection, is primarily a respiratory illness, recent studies showed viral infiltration in GI tissues, suggesting that the digestive tract could serve as an extrapulmonary site for viral replication,^80,81^ provoking symptoms spanning from diarrhea to abdominal pain, vomiting and anorexia.^82^ Extensive research in humans and mouse models proved that SARS-CoV-2 infection leads to gut microbiome dysbiosis over time. Wild-type and SARS-CoV-2 infected cohorts, normalized for antibiotic treatment and sample’s age, and antibiotic-naïve patients showed a loss of alpha diversity, reflecting a shift in the biome composition.^83^ Differences were primarily linked to the enrichment of several taxa.^84,85^ In addition, an increased abundance of opportunistic pathogens known to cause bacteremia, including *Streptococcus*, *Rothia* and *Veillonella*, was detected by shotgun metagenomics in COVID-19 patients compared to healthy controls.^79^ SARS-CoV-2 infected mice were characterized by significant increases of *Akkermasiaceae* and *Proteobacteria* (specifically, *Escherichia* and *Shigella*).^83^ In contrast, the presence of several species of bacteria was negatively correlated with disease severity.^84,85^ Newsome and colleagues further highlighted the disparity between healthy and infected subjects, identifying *Corynebacterium*, *Campylobacter* and *Finegoldia* genera in COVID-19 positive individuals.^86^

Intriguingly, a study led by Lv and colleagues^87^ showed a correlation between the fecal metabolome of infected subjects and gut dysbiosis, with substantial differences between infected and non-infected cohorts. Feces from SARS-CoV-2-positive individuals were enriched in nutrients typically absorbed by the body, such as sucrose, 2-palmitoyl-glycerol, 1,5-anhydroglucitol, and D-pinitol, as well as harmful compounds like oxalate. In contrast, purine metabolites (deoxyinosine, hypoxanthine), behenic acid, D-allose, D-arabinose, and microbe-related products (including 2,4-di-tert-butylphenol) were depleted in SARS-CoV-2-positive patients, reflecting intestinal inflammation and an overall state of malnutrition.^80^ Moreover, SARS-CoV-2 infected mice exhibited an altered gut epithelium, manifested by the increased number of mucus-producing goblet cells, concomitantly with a reduction of Paneth cells. Morphologically, Paneth cells presented phenotypic and genotypic abnormalities. Structurally deformed and misplaced granules were observed, together with a reduced expression of several antimicrobial factors, like lysozyme, defensins, Reg3γ and amyloid A, a condition resembling abnormalities found in IBD patients and indicative of defects in granule packaging and secretion.^83^

## SARS-CoV-2 modulates NLRP3

**Figure 3.**
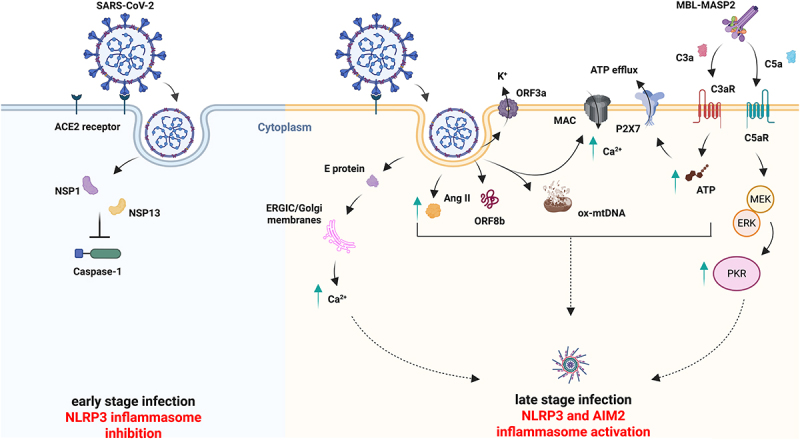
SARS-CoV-2 infection implies NLRP3 inflammasome attenuation at the early stage of infection while necessitating its activation to disseminate at a later stage. Angiotensin-Converting enzyme 2 (ACE2) receptors expressed on the enterocyte’s surface allow SARS-CoV-2 propagation within the gastrointestinal (GI) tract. During early-stage infection, SARS-CoV-2 employes non-structural protein 1 and 13 (NSP1 and NSP13) to deceive the host immune system by dampening the NLRP3 inflammasome activation (left panel). However, viral particles dissemination throughout late-stage infection implies the activation of the NLRP3 inflammasome, encouraging the cytokine storm and triggering systemic inflammation by employing several different strategies. The E protein sustains NLRP3 inflammasome activation through Ca^2+^ mobilization from intracellular stores, whereas the open reading frame 3a (ORF3a) does so via K^+^ efflux. Similarly, angiotensin II (Ang II) cytosolic accumulation activates the inflammasome indirectly. Moreover, active open reading frame 8b (ORF8b) physically interacts with the NLRP3 leucine-rich repeats (LRR) domain to induce its activation. Not surprisingly, late-stage infection is characterized by an active complement cascade, whose intermediates, such as membrane attack complex (MAC), complement Component 3a and 5a (C3a and C5a), converge, leading to the NLRP3 activation. MAC forms plasma membrane pores and generates Ca^2+^ influx. C3a ionophores enhances adenosine triphosphate (ATP) levels and promotes the ATP efflux through the Purinergic receptor P2X, ligand-gated ion channel 7 (P2X7) receptor and, then C5a signals to the mitogen-activated protein kinase (Mek)/extracellular signal-regulated kinase 1/2 (ERK 1/2)/protein kinase R (PKR) pathway, triggering NLRP3 activation. Finally, SARS-CoV-2-induced cytosolic mitochondrial DNA (mtDNA) catalyzes the NLRP3 and absent in melanoma 2 (AIM2) inflammasome activation (right panel).

SARS-CoV-2 dampens NLRP3 inflammasome activation, inducing the immune escape mechanism at the early stage of infection, while it requires activation to propagate later. The virus must evade the immunosurveillance to undergo primary replication at the site of infection before viral dissemination.^88,89^ Viral propagation within the GI district is presumed to be linked to the higher expression of the Angiotensin-Converting Enzyme 2 (ACE2) receptors on the surface of the enterocytes.^90–95^ A recent study, led by Chakraborty and colleagues, showed that the SARS-CoV-2 strategy to evade immunity is through NLRP3 inflammasome inhibition. Viral non-structural protein 1 and 13 (NSP1 and NSP13) significantly inhibit Caspase-1 activity via, the binding to the 40S ribosome subunit and the RNA helicase and nucleoside triphosphatase activities, respectively^95^ although further details are needed to fully elucidate this molecular mechanism. Remarkably, later stages of infection are characterized by canonical NLRP3 inflammasome activation through several approaches,^96^ summarized in Figure 3.

Upon infection, the viral E protein assembles, forming pentameric pores within ERGIC/Golgi membranes to induce cytoplasmic Ca^2+^ mobilization, which ultimately triggers the activation of the NLRP3 inflammasome.^90^ Similarly, the Open Reading Frame 3a (ORF3a) viral protein displays ion channel activity through which it establishes a sufficient K^+^ efflux to provoke the NLRP3 inflammasome activation.^91^ Concurrently, in 2018 Zhao and colleagues showed that the viral S protein-ACE2 signaling pathway competes with the ACE2-angiotensin II (Ang II) axis, which converts Ang II into Ang 1–7 active form, designated to regulate vascular homeostasis and to reduce inflammation. Notably, high intracellular Ang II levels activate activates the NLRP3 inflammasome.^97^ In addition, the Open Reading Frame 8b (ORF8b), the 84-residue peptide formed after the cleavage of ORF8 in the late phase of SARS-CoV-2 infection, binds to the Leucine-rich repeat (LRR) domain of NLRP3, inducing its activation.^92^

The SARS-CoV-2 N protein activates the complement cascade via the mannan-binding lectin (MBL) pathway. MBL then forms a complex with the MBL-associated serine protease 2 (MASP2), which subsequently induces the release of the Complement Component 3a and 5a (C3a and C5a, respectively) and the assembly of the membrane attack complex (MAC).^98–100^ Plasma membrane-inserted MAC promotes Ca^2+^ influx, which is responsible for NLRP3 inflammasome activation.^101^ C3a guides the Adenosine triphosphate (ATP) efflux through the purinergic P2X7 receptor (P2X7R) receptor, inciting inflammasome activation.^102^ At the same time, C5a signals to the mitogen-activated protein kinase (MEK)/extracellular signal-regulated kinase 1/2 (ERK1/2) pathway, which upregulates double-stranded-RNA (dsRNA)-dependent protein kinase R (PKR), also responsible for NLRP3 inflammasome assembly and activation.^103^ Finally, SARS-CoV-2 invasion impairs mitochondrial membranes, causing mitochondrial DNA (mtDNA) to leak into the cytoplasm, an event underpinning NLRP3 and AIM2 inflammasome activation.^104^

Emerging evidence suggests that SARS-CoV-2 infection can influence co-infections with oncogenic pathogens such as EBV and *H. pylori*, potentially exacerbating cancer risk. SARS-CoV-2 has been shown to reactivate latent EBV through stress-induced activation of transcription factors like Activator protein 1 (AP-1) and Specific protein 1 (Sp1), initiating the lytic viral cycle, which promotes inflammation, angiogenesis, and oncogenic signaling pathways. Similarly, *H. pylori* infection may synergize with SARS-CoV-2 by generating reactive nitrogen species such as monochloramine (NH₂Cl), thus activating cellular stress responses and facilitating EBV reactivation. These interactions create a pro-inflammatory microenvironment and immune dysregulation conducive to tumor initiation and progression, particularly in gastric and lymphoid tissues.^105^ Deciphering the intricate crosstalk between SARS-CoV-2, oncogenic viruses like EBV, and bacterial pathogens like *H. pylori* may reveal novel targets to counteract infection-driven tumorigenesis. Furthermore, SARS-CoV-2 exploits IC pathways, as evidenced by the overexpression of inhibitory receptors like PD-1, CTLA4, Tim3, and LAG3, which drive T cell exhaustion and impair immune surveillance.^106^ These mechanisms may compromise antitumor immunity and facilitate cancer progression.

## *H. pylori* and EBV: The missing collaborative link in gut–brain disorders?

Besides gastrointestinal disorders, the gut microbiota can exacerbate neuroinflammation and potentially affect the gut–brain axis, thus contributing to diseases such as Alzheimer’s disease (AD), Parkinson’s disease (PD), and (MS)^107^. However, controversial outcomes are emerging regarding the connection between *H. pylori*, EBV, and MS. Colonization by *H. pylori* and/or EBV is, in fact, also associated with extra-gastric diseases and neuroinflammatory pathways, which might significantly impact the gut–brain axis and potentially lead to severe neurological disorders.^108^ Recent epidemiological studies reported that *H. pylori* infection is correlated with an increased incidence of AD and PD by altering the host immunity, perhaps through the release into the extracellular space of small, spherical bilayer vesicles (20–500 nm in diameter), referred to as *H. pylori* outer membrane vesicles (HP-OMVs).^109^ OMVs carry a variety of macromolecules, such as Lipopolysaccharide (LPS), proteins and nucleic acids, which can activate both innate and acquired immune cells or potentially disrupt the tight junctions of the gastric epithelium, leading to the leaky gut condition; they may even cross the blood–brain barrier (BBB), as shown through *in vivo* experiments.^109^ Thus, in *H. pylori*-infected subjects, once within the systemic circulation, OMVs could target distant districts, including lymphoid organs and the central nervous system (CNS), triggering inflammatory cytokines production through canonical and non-canonical NLRP3 inflammasome activation,^110,111^ which, ultimately, boosts AD and PD onset.^112^ Similarly, abnormal titers of EBV have been observed in the cerebrospinal fluid in subjects with neurological diseases.^113^ Evidence suggests that EBV affects neuronal physiology directly or indirectly via infected B lymphocytes, promoting neuroinflammation and demyelination, ultimately leading to the proliferation, degeneration, and necrosis of glial cells, contributing to nervous system diseases.^114^ Further research is essential to unravel the precise mechanisms by which *H. pylori* and EBV contribute to neurodegeneration and to explore targeted therapies against chronic infections for neurological disease prevention. While studies increasingly support EBV’s involvement in AD, PD, and MS, the link between *H. pylori* and MS remains unclear. The following sections delve deeper into the connections between these pathogens and neurodegenerative diseases.

## EBV infection and AD

**Figure 4.**
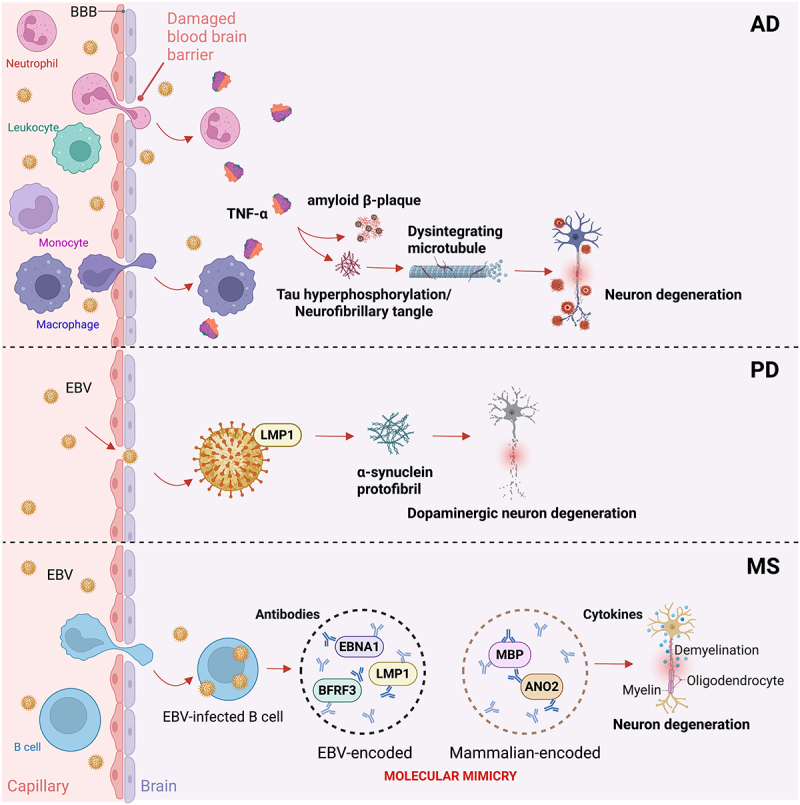
EBV infection contributes to the onset of neurological disorders. EBV infection interferes with the host’s gut–brain axis physiology and may underpin the development of neurological disorders such as Alzheimer’s disease (AD), Parkinson’s disease (PD), and multiple sclerosis (MS). A leaking blood brain barrier (BBB) allows EBV particles to access the brain, where they instigate leukocytes and lymphocytes to produce and secrete tumor necrosis factor-α (TNF-α), which, in turn, is responsible for the hyperphosphorylation of tubulin associated unit (tau) protein, leading to neurofibrillary tangles, and the formation of amyloid β-plaques, thus disintegrating microtubules and accelerating neuron degeneration and the onset of AD (upper panel). Moreover, the viral latent membrane proteins-1 (LMP1) protein carried by infiltrating EBV can lead to dopaminergic neuron degeneration through the interaction between its DPDN sequence and α-syn, causing aggregation of the latter into protofibril structures and subsequently driving the onset of PD (middle panel). Acute EBV infection has also been linked to the onset of MS. Although the mechanisms remain unclear, molecular mimicry between EBV-encoded and mammalian-encoded proteins has recently been proposed as a potential determinant. Virally encoded Epstein–Barr virus nuclear antigen 1 (EBNA1), BamHI fragment rightward open reading frame 3 (BFRF3), and LMP1 share similarities with mammalian proteins myelin basic protein (MBP) and anoctamin 2 (ANO2), thus exposing the latter to the host antibodies and cytokines, which ultimately leads to demyelination, altered axonal electrical conduction, neuron degeneration, and MS development (lower panel).

A damaged BBB allows EBV particles to enter the brain and infect macrophages and neutrophils within the neurological district. Infected leukocytes release IFN-α, TNF-α and cytotoxic factors, such as Natural killer cell granule protein 7 (NKG7), Granzyme A (GZMA) and Beta-2 microglobulin (B2M), which induces, in turn, amyloid β-protein aggregation and hyperphosphorylation of the tubulin associated unit (Tau) protein, thereby promoting the development of AD^115–119^ (Figure 4, upper panel). In addition, it has been shown that EBV-encoded nuclear antigen 2a (BNLF2a) inhibits the transporter associated with antigen processing (TAP) and downregulates Major Histocompatibility Complex (MHC) class I and II expression, leading to an enrichment of the viral polypeptides within the environment.^119^

The virus may also contribute to AD by inducing aberrant cell cycle reentry (CCR) in neurons, a process linked to early neurodegeneration. Through activation of the Rat sarcoma virus oncogene homolog (Ras)-MAPK pathway and modulation of Cyclin D/cyclin-dependent kinase 9 (CDK4), EBV can push quiescent neurons into inappropriate cell cycle phases, leading to mitotic failure and cell death.^120^ A case report described active EBV infection in a patient with Immunoglobulin G antibodies to myelin oligodendrocyte glycoprotein (MOG-IgG)-associated encephalitis who also exhibited AD-like cerebrospinal fluid biomarkers, suggesting a possible link between EBV and the emergence of neurodegenerative signatures.^121^ Together, these findings support a multifactorial role for EBV in AD initiation and progression, involving neuroinflammation, immune evasion, and possibly epigenetic reprogramming of neuronal function.

## EBV infection in PD

The main pathological feature of PD is the degeneration of the dopaminergic neurons due to the formation of abnormal protein aggregates named Lewy bodies. The principal component of these inclusions is α-synuclein (α-syn), which misfolds and accumulates in neuronal cytoplasm. Virally encoded LMP1 cross-reacts with α-syn through its DPDN sequence to induce α-syn aggregation into protofibril structures which promotes PD onset^122^ (Figure 4, middle panel). Moreover, EBV modulates the neuronal cell cycle. The virus dysregulates Cyclin-CDK complexes, particularly CDK-5 and Cyclin E, which are implicated in Lewy body formation and dopaminergic neuron death. Post-mortem PD brains have demonstrated co-localization of EBV markers with α-syn aggregates and evidence of aberrant neuronal cell cycle reentry, suggesting that EBV may drive neurodegeneration through mitotic catastrophe.^120^ In addition, persistent EBV infection activates pro-inflammatory pathways and the subsequent release of IL-1β, IL-6, TNF-α, and C-reactive protein (CRP), thus increasing the risk of PD.^123,124^

An interesting hypothesis is EBV involvement in GI dysmotility. EBV potentially impacts the enteric nervous system (ENS) and might contribute to the early GI manifestations frequently observed in PD, such as delayed gastric emptying. EBV-driven α-syn aggregation in enteric neurons could further disrupt gut motility and reinforce gut–brain axis involvement in PD pathogenesis.^125,126^ However, direct evidence linking EBV to gastrointestinal dysmotility in PD is limited and further studies are needed to support this hypothesis. Williams et al. examined the potential connection between EBV and PD but found no evidence of altered EBV-specific T-cell responses in individuals with PD compared to healthy controls. Thus, EBV is unlikely to drive PD through adaptive T cell immunity.^127^ In a large-scale, observational cohort study using data from two biobanks, Levine et al. confirmed the link between EBV and MS. But, they did not find a direct association of EBV with PD. The study, however, did identify several neurotropic viruses, including influenza and hepatitis, as risk factors for PD, supporting a broader role for viral-induced neuroinflammation in neurodegeneration.^127^ Overall, the evidence of EBV’s involvement in PD remains anecdotal and further research is needed to explore its potential contribution to PD.

## EBV in MS

From the first evidence dating back to 1980, accumulating data have proved a strong positive correlation between elevated titers of EBV and MS onset, compared to healthy individuals.^128–130^ Although the mechanisms of EBV-mediated MS onset remain elusive, molecular mimicry is the best candidate model.^131,132^ Specifically, in-depth studies have demonstrated that serum antibodies from MS patients display a certain degree of cross-reactivity between the EBV-infected B cells secretome, including small capsid protein BamHI fragment rightward open reading frame 3 (BFRF3) and the cytoplasmic protein septin-9,^133^ or EBNA1 and LMP1 with myelin basic protein (MBP), required in the process of nerves myelination.^134,135^ Finally, EBNA1 shares similar features with the chloride-channel protein, anoctamin 2 (ANO2), which is associated with electrical conduction in axons^136,137^ (Figure 4, lower panel). Thus, it has been proposed that cytokines and autoantibodies derived from active B cells might induce neurodegeneration by tricking the immune system into attacking the body’s tissues. However, other unknown mechanisms cannot be excluded.

## *H. pylori* and AD

Noori and colleagues showed that clinically relevant *H. pylori* strains enhanced the expression of the primary AD-associated risk genes, such as Apolipoprotein E (ApoE) ε4 (*ApoE4*), while reducing protective counterparts (e.g., Apolipoprotein E2, ApoE2), and, hence, promoting neuroinflammation and cognitive impairment.^138–141^ Concomitantly, a recent *in-vitro* study, led by Palacios and coauthors, demonstrated that OMVs could revise the astrocyte’s levels of the intermediate filament glial fibrillary acidic protein (GFAP), vimentin, plasma membrane α_V_β_3_ integrin, hemichannel connexin 43 and promote the release of IFN-γ via NF-κB activation. Thus, the astrocytes can be switched to an unequivocal neurotoxic phenotype,^109^ which is usually a hallmark of neuroinflammation leading to AD and almost every other neurodegenerative condition.^142^

**Figure 5.**
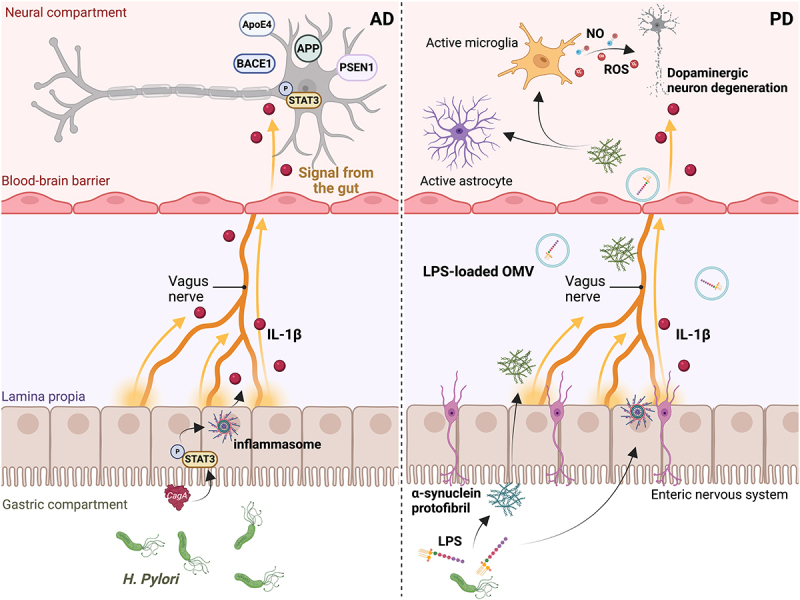
Acute *H. pylori* infection promotes the onset of neurological disorders through inflammasome activation. Persistent *H. pylori* colonization might underly the onset and development of Alzheimer’s disease (AD) and Parkinson’s disease (PD). Cytotoxin-associated gene a (CagA)- signal transducer and activator of transcription 3 (STAT3)-inflammasome axis engagement results in the secretion of mature IL-1β, reaching the brain district through the vagus nerve and supporting the expression of AD-associated markers, such as Apolipoprotein E4 (ApoE4), amyloid β-precursor protein (APP), beta-site APP cleaving enzyme 1 (BACE1), and presenilin-1 (PSEN1), left panel. Moreover, besides its function in inducing inflammasome activation, LPS sustains α-syn conformational changes into protofibril structures, which, once within the neural compartment, prompt activated astrocytes and microglia to release nitric oxide (NO) and reactive oxygen species (ROS), ultimately damaging dopaminergic neurons, causes of PD onset.

Notably, these OMVs have been shown to penetrate neural tissue and co-localize with amyloid-β plaques, exacerbating amyloid deposition, synaptic damage, and cognitive decline in AD mouse models.^143^ Mechanistically, CagA induces the secretion of pro-inflammatory cytokines via Signal transducer and activator of transcription 3 (STAT3)-dependent inflammasome activation, which, once within the neural compartment, presumably through the vagus nerve, promotes the nuclear translocation of phosphorylated STAT3, leading to enhanced levels of AD-associated markers, including ApoE4, amyloid beta precursor protein (APP), beta-site APP cleaving enzyme 1 (BACE1), and Presenilin-1 (PSEN1) (Figure 5, left panel). While CagA is a well-known virulence factor of *H. pylori* associated with gastric diseases, there is currently a lack of direct evidence for its presence in neural tissue in the context of AD. However, CagA has been shown to disrupt cellular signaling pathways, and its potential role in neurodegeneration warrants further investigation.

## *H. pylori* infection in PD

*H. pylori* adopts several strategies to promote the onset of PD. The lipid component of the LPS induces conformational changes in α-syn, a key protein involved in PD pathophysiology, converting it into protofibril structures, which transit through the ENS and vagus nerve. In addition to its neuroinflammatory effects, *H. pylori* has been linked to gastric dysmotility and delayed gastric emptying in PD patients. *H. pylori* worsens non-motor symptoms, may impair levodopa absorption, and contributes to the overall disease burden. These early gastrointestinal manifestations are believed to stem from *H. pylori*-induced mucosal inflammation and its effects on the ENS.^144^ Once within the brain, these aggregates activate astrocytes and microglia, which, in turn, release nitric oxide (NO) and Reactive oxygen species (ROS), following dopaminergic neuron degeneration, an explicit feature of PD progression. Moreover, LPS-loaded OMVs, by crossing the BBB, promptly reach the neuronal compartment and convert astrocytes and microglia into their reactive phenotypes. Finally, inflammasome-derived IL-1β contributes to neuroinflammation and PD onset^145^ (Figure 5, right panel).

## SARS-CoV-2 infection alters the gut–brain axis, inducing neuroinflammation and neurological symptoms

**Figure 6.**
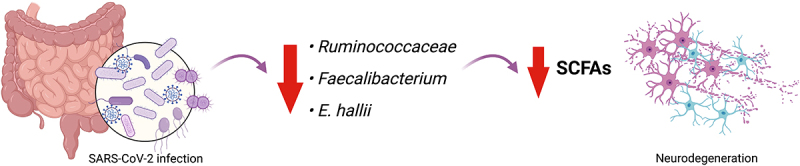
SARS-CoV-2 infection leads to gut dysbiosis, reduction of SCFAs-producing bacteria and neurodegeneration. SARS-CoV-2-infected people show a significant decrease of SCFAs-producing bacterial species, such as *Ruminococcaceae*, *Faecalibacterium* and *Eubacterium hallii* (*E. hallii*), whose loss is responsible for neurodegeneration and brain issues.

A growing body of research has established a connection between gut–brain issues and SARS-CoV-2 infection. Roughly 66% of affected subjects develop neurological symptoms worsened by gut dysbiosis.^146^ It has been observed that SARS-CoV-2 patients display a significant reduction in short-chain fatty acids (SCFA)-producing bacteria like *Ruminococcaceae*, *Faecalibacterium*, and *Eubacterium hallii* (*E. hallii*), which play roles in brain inflammation.^147^ In addition, due to SARS-CoV-2-induced blood-intestinal barrier disruption, factors like LPS, IL-1β, IL-6, TNF-α and microbial metabolites reach neural tissues causing inflammation (Figure 6). SARS-CoV-2-infected individuals with GI symptoms have been found to have higher levels of IL-6 and fecal calprotectin, suggesting gut inflammation and compromised gut integrity.^148^ Through an *in vivo* model, Albornoz and colleagues showed that SARS-CoV-2 can bind and enter microglia, promoting a robust microglial NLRP3 inflammasome activation in an ACE2-dependent manner, further enhanced in the presence of α-syn fibrils. Treatment with the specific NLRP3 inflammasome inhibitory drug, MCC950, entirely ablated IL-1β secretion, significantly attenuating neurological symptoms.^149^

## Clinical trials for pathogen-associated GI inflammation and cancer

We searched the ClinicalTrials.gov database for the most recent clinical trials conducted over the last 5 y (2020–2025) investigating gastrointestinal inflammation and cancers associated with EBV, *H. pylori*, and SARS-CoV-2 (Table 1). In EBV-associated gastrointestinal malignancies, many of these trials emphasize immunotherapy-based approaches, such as NCT05535569^150^ and NCT03755440,^151^ which examine IC inhibitors, including anti-PD-L1 (nivolumab) and PD-1 antibodies (camrelizumab), respectively. The NCT05535569 clinical trial assessed the efficacy of nivolumab combined with paclitaxel in patients with EBV-related microsatellite instability-high/deficient mismatch repair (MSI-H/dMMR) or PD-L1–positive advanced gastric cancer (AGC) which had progressed after first-line platinum and fluoropyrimidine chemotherapy. The results highlight EBV status as a crucial biomarker that may influence the response to immunotherapy in this context. The study’s findings are reinforced by Cancer Genome Atlas studies, which identified EBV-associated tumors exhibiting elevated PD-L1 expression and microsatellite unstable tumors responsive to immunotherapy subtypes.^150^

**Table 1.** Clinical trials for pathogen-associated GI inflammation and cancer.

Clinical Trials.gov ID	Phase	Title	Results	References
NCT05535569	Ib/II	Evaluation of the Safety and Efficacy of Nivolumab in Combination With Paclitaxel in Epstein–Barr Virus(EBV)-Related, or Microsatellite Instability-High (MSI-H), or Programmed Cell Death Ligand 1 (PD-L1) Positive Advanced Gastric Cancer	**Median Progression-Free Survival (PFS)**: 4.4 months (95% CI 1.8-NA^1^) **Median Overall Survival (OS)**: 5.8 months (95% CI 4.8-NA) **Objective Response Rate (ORR)**: Not specified for EBV group **Genomic findings**: No significant correlation with survival, but EBV+ tumors showed high PD-L1 expression. HLA-A02 supertype was associated with better response to ICIs **Cytokine analysis findings**: Higher baseline IL-1Ra, IL-6 and IL-8 levels correlated with poorer outcomes **Conclusion for EBV-associated AGC treatment**: Additional biomarkers and combination therapies are needed to optimize response and survival outcomes for this subgroup.	*Lee CK et al*. *Gastric Cancer, 2024.^150^*
NCT03755440	II	PD-1 Antibody in EBV Positive Metastatic Gastric Cancer Patients.	**Median Pogression-Free Survival (PFS)**: 2.2 months (95% CI 1.4-NR^2^) **Median Overall Survival (OS)**: 6.8 months (95% CI 1.7-NR) **Objective Response Rate (ORR)**: 0% (No patient achieved CR^3^ or PR^4^) **Genomic findings**: most commonly mutated genes were PIK3CA, KMT2D, FAT1, and ARID1A **Cytokine analysis findings**: Low PD-L1 expression in 5/6 patients; TMB was low **Conclusion for EBV-associated AGC treatment**: none of the tumors fully disappeared or shrank by at least 30% with camrelizumab treatment.	*Sun YT et al. Am J Cancer Res. 2021.^151^*
*NCT06228963*	*II*	Orelabrutinib in the Treatment of HP-positive Gastric MALT Lymphoma	Not yet available	*https://clinicaltrials.gov/study/NCT06228963.*
*NCT04660123*	*IV*	Helicobacter Pylori Eradication With Berberine Plus Amoxicillin Triple Therapy Versus Bismuth-containing Quadruple Therapy	**Eradication rate of primary therapy**: 93,3% **Eradication rate of rescue therapy**: 75,8% **Highest eradication rate (98,4%)**: therapy with Lansoprazole **Conclusion**: Bismuth quadruple therapy is highly effective for *H. Pylori* eradication. Proton Pump Inhibitor (PPI), Lansoprazole, raises gastric pH and increases the effectiveness of antibiotics.	*Zhu X et al*. *Chin Med J 2023^152^*
NCT04697186	*IV*	Helicobacter Pylori Eradication With Berberine Plus Amoxicillin Triple Therapy Versus Bismuth-containing Quadruple Therapy	**Eradication rate**: 80% **Adverse events**: mild or moderate **Berberine triple therapy vs bismuth‐containing quadruple therapy**: no statistical difference in eradication	*Chen XX et al*. *J Dig Dis. 2022. ^153^*
NCT05800080	*N/A*	An Exploratory Study of Immunotherapy Combined With Anlotinib and Chemotherapy in Perioperative Treatment of LAGC	**Not yet recruting**	*https://clinicaltrials.gov/study/NCT05800080.*
*NCT04858425*	*II*	Safety and Efficacy of Niclosamide in Patients With COVID-19 With Gastrointestinal Infection (RESERVOIR)	**Viral clearance**: 23% on day 43 **Clinical results**: not available	*https://clinicaltrials.gov/study/NCT04858425.*
*NCT04420676*	*N/A*	Synbiotic Therapy of Gastrointestinal Symptoms During COVID-19 Infection (SynCov)	**Alterations in gut microbiome associated with SARS-CoV-2 infection**: increase in the Bacteroidetes taxa and a decrease in the Christensenellaceae and Ruminococcaceae taxa **Conclusions of probiotics use in COVID-19 patients**: significantly modulated microbiome beta diversity (*p* = 0.001), introduced the Enterococcus faecium W54 strain; symptoms, COVID-19-related taxa, and the fecal metabolome were not affected	*Horvath A et al*. *Nutrients 2024. ^161^*

^1^NA or N/A: Not Available or Not Applicable (there were insufficient number of events to calculate a reliable estimate); ^2^NR: Not Reached (not enough patients experienced the event, e.g., disease progression or death). ^3^CR: Complete Response: no detectable cancer remains. ^4^PR: Partial Response: at least 30% decrease in the diameters of target lesions (tumors), ^5^beta diversity: differences in gut microbiome composition between SARS-CoV-2 infected patients and healthy controls, probiotic-treated and placebo groups.

Future treatment strategies should integrate additional biomarkers and combination therapy to optimize treatment for EBV-associated AGC patients. The results from the NCT03755440^151^ clinical trial showed that EBV positivity alone is not a sufficient predictor of response to camrelizumab in metastatic gastric cancer. PD-L1 expression, Tumor Mutation Burden (TMB), and other biomarkers may be more relevant for guiding immunotherapy decisions in this subgroup. Targeted treatments are also being tested for *H. pylori*-related conditions, including NCT06228963, which evaluates orelabrutinib, a Bruton’s tyrosine kinase (BTK) inhibitor, targeting B-cell receptor (BCR) signaling and safety for the treatment of *H. pylori*-positive gastric MALT lymphoma. BTK inhibitors are used to treat B-cell tumors (e.g., diffuse large B-cell lymphoma, DLBCL). This clinical trial started in 2024 at Fudan University and consists of a triple therapy for *H. pylori* plus orelabrutinib; thus, the results are unavailable. Another common strategy is *H. pylori* eradication (NCT04660123 and NCT04697186), which improves the effectiveness of infection elimination.^152,153^ These studies show a high eradication rate for both berberine triple therapy and bismuth-containing quadruple therapy with no statistically significant difference between them.

In addition to these interventions, current therapeutic approaches for *H. pylori*-related gastric pathology also focus on patients with precancerous lesions and established malignancy. *H. pylori* eradication remains the cornerstone for treating atrophic gastritis and intestinal metaplasia, potentially reversing or halting lesion progression.^154,155^ High-risk individuals are advised to undergo surveillance endoscopy and make lifestyle changes.^156^ For patients with gastric adenocarcinoma, treatment options include surgical resection, endoscopic mucosal dissection, and combination chemotherapy. Information on gastric cancer prevention, diagnosis, treatment, and supportive care for clinicians and patients can be found in PDQ (Physician Data Query), an evidence-based database.^157^ Shitara et al.’s exploratory analysis of the phase III KEYNOTE-061 trial showed that high T-cell-inflamed gene expression predicted a better response to pembrolizumab. At the same time, elevated myeloid-derived suppressor cell (mMDSC) signatures were linked to poorer outcomes. This highlights the potential for combining immunotherapy with myeloid-targeting strategies.^158^ Targeted therapies showed promising outcomes in advanced unresectable or metastatic gastric or gastro-esophageal junction adenocarcinoma, such as trastuzumab for Human Epidermal growth factor Receptor 2 (HER2)-positive tumors and zolbetuximab, a monoclonal antibody targeting claudin-18 isoform 2 (Claudin 18.2)-positive cancers, alongside IC inhibitors like nivolumab.^159,160^

Another study, NCT05800080, explores the combination of chemotherapy, immunotherapy, and targeted therapy to identify better treatments for advanced stomach cancer. This study is not yet recruiting patients so the results are unavailable. Although terminated, the NCT04858425 study highlights an interest in understanding how infections affect gastrointestinal health. The NCT04420676^161^ clinical study examines the potential of probiotics as a therapeutic option. It revealed that while probiotic treatment altered patients’ microbiome beta diversity, it did not alleviate gastrointestinal symptoms or impact COVID-19-related taxa and the fecal metabolome. These findings indicate that probiotics can affect gut microbiome composition during SARS-CoV-2 infection, but their therapeutic effect on gastrointestinal symptoms remains limited. These trials reflect the increasing recognition that pathogens significantly contribute to GI inflammation and malignancies, prompting further research into underlying mechanisms and potential treatments. Current trials face challenges such as small sample sizes, incomplete results, and the limitations of single biomarkers, which hinder their overall impact. Future studies should utilize multi-omics approaches, larger cohorts, and combination therapies to improve immunotherapy, targeted treatments, and microbiome-based interventions for pathogen-associated GI diseases.

## Conclusion

The intricate interplay between pathogens such as EBV, *H. pylori*, SARS-CoV-2, and the GI microenvironment underscores the significant role of infectious agents in driving chronic inflammation and carcinogenesis. These pathogens employ diverse mechanisms, including immune evasion, epigenetic modulation, and disruption of host cellular signaling, to establish persistent infections and foster oncogenic microenvironments. Understanding these mechanisms advances our knowledge of GI diseases and paves the way for innovative diagnostic and therapeutic approaches. EBV and *H. pylori* remain major contributors to GI malignancies, with synergistic effects in co-infection scenarios suggesting a heightened risk of gastric transformation. Emerging evidence implicates viral and bacterial interactions in reshaping the tumor microenvironment, supporting immune escape, and facilitating oncogenic signaling pathways such as NF-κB activation and DNA methylation. Similarly, the role of SARS-CoV-2 in altering gut microbiota and inducing systemic inflammation provides new insights into the gut–lung axis and the potential long-term impacts of COVID-19 on GI health. These findings highlight the critical need to consider infectious agents in the context of chronic GI inflammation and malignancies.

## Future perspectives

The future of GI disease management lies in exploiting the pathogen-host interactions to develop targeted therapies. Several promising avenues for research and intervention emerge from this review. For instance, advances in molecular profiling offer the potential to identify pathogen-specific biomarkers, such as viral miRNAs or epigenetic signatures. These could improve the early detection of GI malignancies and the stratification of high-risk patients. The immune evasion strategies employed by EBV, *H. pylori*, and SARS-CoV-2 underscore the importance of developing therapies that enhance immune surveillance. Vaccines targeting these pathogens could be pivotal in preventing infection-driven GI inflammation and cancer. Understanding the molecular pathways these pathogens exploit, such as PD-L1 upregulation in EBV-associated cancers or CagA-induced signaling in *H. pylori*-related diseases, opens the door for precision-targeted therapies. Combinations of IC inhibitors, demethylating agents, and RNA-based therapies could significantly improve patient outcomes.

The role of gut dysbiosis in disease progression highlights the need for therapies to restore microbial balance. Probiotics, prebiotics, and microbiota transplantation are promising strategies that warrant further investigation. To unravel the complex dynamics of co-infections and their contributions to disease progression, there is a pressing need for longitudinal studies and advanced co-infection models replicating real-world scenarios. Addressing these research gaps will advance our understanding of infection-driven GI diseases and provide a framework for developing novel, effective, personalized therapeutic strategies. As the prevalence of GI malignancies continues to rise globally, the insights gained from studying the roles of EBV, *H. pylori*, and SARS-CoV-2 will be instrumental in reducing the burden of these diseases and improving patient outcomes.

## Author agreement

all authors have seen and approved the final version of the submitted manuscript.

## Abbreviations

4C: Chromosome Conformation Capture-on-Chip
α-syn: α-synuclein
ACE2: Angiotensin-Converting Enzyme 2
AD: Alzheimer disease
AGC: advanced gastric cancer
AIM2: Absent in melanoma 2
AKT: Protein kinase B
Ang II: Angiotensin II
ANO2: Anoctamin 2
AP-1: Activator protein 1
APC: Antigen-presenting cell
ApoE: Apolipoprotein E
ApoE2: Apolipoprotein E2
ApoE4: Apolipoprotein E ε4
APP: Amyloid beta precursor protein
ARID1A: AT-rich interaction domain 1A
ATP: Adenosine triphosphate
B2M: Beta-2 microglobulin
babA: Blood group antigen binding adhesin
BACE1: Beta-site APP cleaving enzyme 1
BamHI: Bacillus amyloliquefaciens H 1 strain
BARF0: B cell receptor-associated factor 0
BBB: blood brain barrier
Bcl2: B-cell leukemia/lymphoma 2 protein
BCR: B-cell receptor
BFRF3: BamHI fragment rightward open reading frame 3
BMRF1: Recombinant EBV DNA polymerase processivity factor
BNLF2a: EBV-encoded nuclear antigen 2a
BTK: Bruton’s tyrosine kinase
BTLA: B and T lymphocyte attenuator
BZLF1: BamHI Z fragment leftward open reading frame 1
C3a: Complement Component 3a
C5a: Complement Component 5a
CagA: Cytotoxin-associated gene A
CCR: cell cycle reentry
CD: Cluster of differentiation
CD4^+^: Cluster of differentiation 4
CD8: Cluster of differentiation 8
CD11b: Cluster of differentiation 11 b
CD18: Cluster of differentiation 18
CD21: Complement receptor type 2
CD47: Cluster of differentiation 47
CD69: Cluster of differentiation 69
CDK4: Cyclin-dependent kinase 4
CDK5: Cyclin-dependent kinase 5
CDK9: Cyclin-dependent kinase 9
ChIP: Chromatin Immunoprecipitation Sequencing
CI: Confidential interval
CIMP: CpG island methylator phenotype
CMV: Cytomegalovirus
CNS: central nervous system
COVID-19: Coronavirus disease 2019
CpG: Cytosine phosphate diester guanine
CRP: C-reactive protein
CTLA4: Cytotoxic T-lymphocyte associated protein 4
CTLs: Cytotoxic T-lymphocytes
DAPK3: Death-associated protein kinase 3
DCs: Dendritic cells
DLBCL: Diffuse large B-cell lymphoma
Dmmr: deficient mismatch repair
DNMT3B: DNA methyltransferase 3 beta
dsRNA: double-stranded RNA
*E. hallii*: *Eubacterium hallii*
EBERs: EBV-encoded small RNAs
EBNA1: Epstein–Barr virus nuclear antigen 1
EBNA2: Epstein–Barr virus nuclear antigen 2
EBNA3C: Epstein–Barr virus nuclear antigen 3C
EBV: Epstein–Barr virus
EBVaGC: EBV-associated gastric cancer
EBVnGC: EBV-non-associated GC
EC: Esophageal cancer
EMT: Epithelial-Mesenchymal Transition
ENS: enteric nervous system
EOC: Epithelial ovarian cancer
ERK1/2: Extracellular signal-regulated kinase ½
ETEC: Enterotoxigenic E. Coli
FAT1: FAT atypical cadherin 1
GC: Gastric cancer
GFAP: Glial fibrillary acidic protein
gH/gL/gp42: H/glycoprotein L/gp42
GI: Gastrointestinal
gp42: Glycoprotein 42
GZMA: Granzyme A
*H. Felis*: *Helicobacter felis*
Hi-C: High-throughput Chromosome Conformation Capture chromosome conformation capture
*H. pylori*: *Helicobacter pylori*
HER: Human Epidermal growth factor Receptor 2
HLA: Human leukocyte antigens
HP-OMVs: *H. pylori* outer membrane vesicles
HSP70: Heat shock protein 70
IAP: Integrin-associated protein
IARC: International Agency for Research on Cancer
IBD: Inflammatory bowel disease
IC: Immune checkpoint
ICOSL: Inducible T-cell co-stimulator ligand
IDO1: Indoleamine 2,3-dioxygenase 1
IGF2: Insulin-like Growth Factor 2
IgG: Immunoglobulin G
IHC: Immunohistochemistry
IL-1α: Interleukin-1 alpha
IL-1β: Interleukin-1 beta
IL-1Ra: Interleukin-1 receptor antagonist
IL-5: Interleukin-5
IL-6: Interleukin-6
IL-8: Interleukin-8
IL-10: Inteleukin-10
IL-12: Interleukin-12
IL-18: Interleukin-18
IL-23: Inteleukin-23
IFN-α: Interferon-alpha
IFN-γ: Interferon-gamma
IRF-3: Interferon regulatory factor-3
ITIM: Immunoreceptor tyrosine-based inhibitor motif
JAK2: Janus Kinase 2
KMT2D: Lysine methyltransferase 2D
LAG3: Lymphocyte activation gene 3
LINE-1: Long INterspersed Element 1
LMP1: latent membrane protein-1
LMP2A: latent membrane protein-2A
LMPs: Latent membrane proteins
LPS: Lipopolysaccharide
LOI: Loss of imprinting
LRR: Leucine-rich repeat
MAC: Membrane attack complex
MALT: Mucosa-associated lymphoid tissue
MAPKs: Mitogen-activated protein kinases
MASP2: MBL-associated serine protease 2
MBL: Mannan-binding lectin
MBP: Myelin basic protein
MEK: Mitogen-activated protein kinase
miRNAs: MicroRNAs
MHC: Major histocompatibility complex
mMDSC: myeloid-derived suppressor cell
MOG: myelin oligodendrocyte glycoprotein
MN: Mononuclear
mtDNA: Mitochondrial DNA
MS: Multiple sclerosis
MSI-H: microsatellite instability-high
NF-Κb: Nuclear factor kappa-light-chain-enhancer of activated B cells
NK: Natural killer
NKG7: Natural killer cell granule protein 7
NLRP3: Nucleotide-binding domain, leucine-rich – containing family, pyrin domain – containing-3
NO: Nitric oxide
NPC: Nasopharyngeal carcinoma
NSP1: Non-structural protein 1
NSP13: Non-structural protein 13
ORF3a: Open Reading Frame 3a
ORF8b: Open Reading Frame 8b
ORR: Overall responsive rate
OS: Overall survival
P2X7R: Purinergic P2×7receptor
PBMCs: Peripheral blood mononuclear cells
PCNA: Proliferating cell nuclear antigen
PD: Parkinson’s disease
PD-1: Programmed Cell Death Protein 1
PD-L1: Programmed Death-Ligand 1
PD-L2: Programmed Death-Ligand 2
PDQ: Physician Data Query
PFS: Progression-free survival
PIK3CA: Phosphatidylinositol-4,5-Bisphosphate 3-Kinase Catalytic Subunit Alpha
PKC: Protein kinase C
PKR: Protein kinase R
PMN: Polymorphonuclear
PPI: Proton pump inhibitor
pRB: retinoblastoma protein
PSEN1: Presenilin-1
PTEN: Phosphatase and tensin homolog protein
qPCR: Quantitative polymerase chain reaction
Ras: Rat sarcoma virus oncogene homolog
ROS: Reactive oxygen species
SARS-CoV-2: Severe acute respiratory syndrome coronavirus 2
SCFA: Short-chain fatty acids
SHP-2: Src homology two domain-containing protein tyrosine phosphatase-2
SIBO: Small Intestinal Bacterial Overgrowth
SIRPα: Signal regulatory protein alpha
SNPs: Single-nucleotide polymorphisms
Sp1: Specific protein 1
Src: Sarcoma virus
STAT3: Signal transducer and activator of transcription 3
T4SS: type IV secretion system
TAB1: TGF-beta-activated kinase 1 (TAK1) binding protein 1
TAK1: TGF-beta-activated kinase 1
TAP: Transporter associated with antigen processing
Tau: Tubulin associated unit
TBK-1: TANK-binding kinase-1
Th1: T Helper 1
Th2: T helper 2
TIGIT: T cell immunoreceptor with Ig and the ITIM domain
TIM3: T-cell immunoglobulin and mucin domain 3
TMB: Tumor Mutation Burden
TME: Tumor immune microenvironment
TNF-α: Tumor necrosis factor alpha
TRM: tissue-resident memory T
p53: Tumor protein P53
VacA: Vacuolating cytotoxin gene A
VS: Virological synapses
Zta: Zebra Trans-Activator.
